# Genetic determinants of serum 25-hydroxyvitamin D concentration during pregnancy and type 1 diabetes in the child

**DOI:** 10.1371/journal.pone.0184942

**Published:** 2017-10-04

**Authors:** Maija E. Miettinen, Melissa C. Smart, Leena Kinnunen, Valma Harjutsalo, Linnea Reinert-Hartwall, Irene Ylivinkka, Heljä-Marja Surcel, Christel Lamberg-Allardt, Graham A. Hitman, Jaakko Tuomilehto

**Affiliations:** 1 Chronic Disease Prevention Unit, National Institute for Health and Welfare, Helsinki, Finland; 2 Blizard Institute, Barts and the London School of Medicine and Dentistry, Queen Mary University of London, London, United Kingdom; 3 University of Essex, Essex, United Kingdom; 4 Genomics and Biomarkers Unit, National Institute for Health and Welfare, Helsinki, Finland; 5 Folkhälsan Institute of Genetics, Folkhälsan Research Center, Helsinki, Finland; 6 Abdominal Center Nephrology, University of Helsinki and Helsinki University Hospital, Helsinki, Finland; 7 Research Program Unit, Diabetes and Obesity, Helsinki, Finland; 8 Vaccination Programme Unit, National Institute for Health and Welfare, Helsinki, Finland; 9 Translational Cancer Biology Research Program, University of Helsinki, Helsinki, Finland; 10 Impact Assessment Unit, National Institute for Health and Welfare, Oulu, Finland; 11 Department of Food and Environmental Sciences, Helsinki, Finland; 12 Center for Vascular Prevention, Danube-University Krems, Krems, Austria; 13 South Ostrobothnia Central Hospital, Seinäjoki, Finland; 14 Diabetes Research Group, King Abdulaziz University, Jeddah, Saudi Arabia; 15 Dasman Diabetes Institute, Dasman, Kuwait; Medical University of Gdańsk, POLAND

## Abstract

**Objective:**

The *in utero* environment plays an important role in shaping development and later life health of the fetus. It has been shown that maternal genetic factors in the metabolic pathway of vitamin D associate with type 1 diabetes in the child. In this study we analyzed the genetic determinants of serum 25-hydroxyvitamin D (25OHD) concentration during pregnancy in mothers whose children later developed type 1 diabetes and in control mothers.

**Study design:**

474 mothers of type 1 diabetic children and 348 mothers of non-diabetic children were included in the study. We previously selected 7 single nucleotide polymorphisms (SNPs) in four genes in the metabolic pathway of vitamin D vitamin based on our previously published data demonstrating an association between genotype and serum 25OHD concentration. In this re-analysis, possible differences in strength in the association between the SNPs and serum 25OHD concentration in mothers of type 1 diabetic and non-diabetic children were investigated. Serum 25OHD concentrations were previously shown to be similar between the mothers of type 1 diabetic and non-diabetic children and vitamin D deficiency prevalent in both groups.

**Results:**

Associations between serum 25OHD concentration and 2 SNPs, one in the vitamin D receptor (*VDR*) gene (rs4516035) and one in the group-specific component (*GC*) gene (rs12512631), were stronger during pregnancy in mothers whose children later developed type 1 diabetes than in mothers whose children did not (*p*_interaction_ = 0.03, 0.02, respectively).

**Conclusions:**

We show for the first time that there are differences in the strength of genetic determinants of serum 25OHD concentration during pregnancy between the mothers of type 1 diabetic and non-diabetic children. Our results emphasize that the *in utero* environment including maternal vitamin D metabolism should be important lines of investigation when searching for factors that lead to early programming of type 1 diabetes.

## Introduction

Type 1 diabetes is an autoimmune disease where the insulin-producing β-cells in the pancreas are destroyed leading to a life-long external insulin dependency. Type 1 diabetes is one of the most common chronic diseases in children, but it can be diagnosed at any age [1,2]. Type 1 diabetes is a result of complex interplay between genes and environment. The fast raise in the incidence of type 1 diabetes especially in the western world during the past decades [3] emphasize the role of the environmental factors in the development of the disease. Environmental triggers that have led to this increase have not been revealed [4].

According to the “Barker Hypothesis”, also referred to as the “Fetal Origin Hypothesis”, diseases occurring in different stages of life may have their origin in the fetal or neonatal environment [5]. The fact that type 1 diabetes-related autoantibodies that precede the diagnosis can be detected sometimes only months after birth [6], suggests fetal programming of type 1 diabetes. Also, maternal infections such as rubella and enterovirus infections [7,8], high maternal age and high birthweight of the infant have been associated with an increased risk for the disease [9,10].

Vitamin D is produced in the skin through sunlight exposure and obtained from dietary sources and supplements. It regulates mineral and bone metabolism and has various functions in the immune system [11]. 25-hydroxyvitamin D (25OHD) is the major circulating vitamin D metabolite and a good indicator of the vitamin D status of a person [12].

For decades, vitamin D has been suspected to modify the risk for type 1 diabetes. The first indication was that type 1 diabetes tends to be more common in areas with less sunlight and thus lower amounts of vitamin D produced in the skin [13]. Experiments in a murine model of type 1 diabetes, non-obese diabetic (NOD) mice, strengthened the link between vitamin D and type 1 diabetes. In the NOD-mice vitamin D deficiency significantly increases the incidence of diabetes and administration of vitamin D is able to protect from the disease [14].

Low maternal serum 25OHD concentration or inadequate intake of vitamin D from food or supplements during pregnancy or infancy has been associated with an increased risk for type 1 diabetes in the child, but the results have been partly conflicting [15–22]. The inconsistency may result from the fact that the amount of vitamin D supplementation may have been too low to produce significant effects (mainly 10 μg/day) [23]. An exception is a Finnish study where a daily dose of 50 μg of vitamin D supplementation was recommended (in 1960s) for the infants [15] leading presumably to a marked difference in vitamin D status between the supplement users and the non-users. In the Finnish study it was shown that the risk for type 1 diabetes was reduced by 88% in children who received vitamin D supplementation regularly compared with those who did not receive vitamin D supplementation.

In our previous study we did not see difference in serum 25OHD concentrations during the first trimester of pregnancy between Finnish mothers whose children later on developed type 1 diabetes, compared with mothers whose children did not [17]. In a similar study setting in Norway, higher maternal serum 25OHD concentration during the last trimester of pregnancy associated with a decreased risk for type 1 diabetes in the offspring [16]. Vitamin D deficiency was more prevalent among pregnant women in Finland than in Norway, although the methodological differences in vitamin D analytics need to be taken into account when comparing the results.

Genetic factors are known to modify serum 25OHD concentration. The heritability of serum 25OHD concentration has been estimated to be at least 30% [24,25]. Several single nucleotide polymorphisms (SNPs) in the metabolic pathway of vitamin D contribute in the genetic component of serum 25OHD concentration [26–31].

SNPs in the metabolic pathway of vitamin D have been associated also with type 1 diabetes [32–37]. For example, SNPs in NAD synthetase 1/ 7-dehydrocholesterol reductase (*NADSYN1/DHCR7)* gene locus and SNPs in *CYP27B1*, *CYP2R1* genes and have been associated with type 1 diabetes or with type 1 diabetes related autoantibodies [33,35,38]. In the metabolic pathway of vitamin D, *NADSYN1/DHCR7* controls the availability of 7-dehydrocholesterol that is needed for the vitamin D synthesis in the skin and *CYP2R1* encode (along with *CYP27A1)* enzymes that produce 25OHD. In the circulation, 25OHD is bound to the vitamin D binding protein (DBP) (encoded by the group-specific component *GC* gene) and hydroxylated to the active form of vitamin D, 1,25-dihydroxyvitamin D (1,25OHD), by *CYP27B1*. The active form of vitamin D acts through the vitamin D receptor (VDR) encoded by the *VDR* gene. The *VDR* regulates the expression of at least 500 genes [39].

We found earlier that the genotype frequency of certain SNPs in the *VDR* gene differed between mothers of type 1 diabetic and non-diabetic children [40]. We found also that certain SNPs in genes in the metabolic pathway of vitamin D associate with serum 25OHD concentration in all pregnant mothers analyzed as one group [40]. In this study we have re-analysed the data and report differences in the strength of genetic associations between serum 25OHD concentration and SNPs in the metabolic pathway of vitamin D in mothers of type 1 diabetic and non-diabetic children.

## Materials and methods

### Study population

A detailed description of the study population has been previously published [17,40]. Briefly, 751 families with type 1 diabetic child and 751 families with non-diabetic child of same age were invited to participate in the study. Serum samples were derived from the Finnish Maternity Cohort (FMC). The FMC collects serum samples from almost all (98%) pregnancies in Finland during the end of first trimester of pregnancy. Serum samples used in the present study were collected during 1993–2000.

In this study we included all mothers (474 mothers of type 1 diabetic children and 348 mothers of non-diabetic children) that had both SNP genotyping and serum 25OHD concentration results available. The problem of possible uneven distribution of sample collection month between mothers of type 1 diabetic and non-diabetic children was addressed by including sample collection month in the model in the analyses. Written informed consent was collected from all women. The ethics committee of the Hospital District of Helsinki and Uusimaa approved the study.

### DNA extraction, SNP selection and genotyping

Saliva samples were collected using Oragene kits (Oragene Inc., Ottawa, Ontario, Canada) for DNA extraction. The DNA extraction and the genotyping have been described in detail in our previously published study [40]. Briefly, DNA was isolated using Oragene kits (DNA Genotek, Ottawa, Ontario, Canada). Genotyping was done using TaqMan (Applied Biosystems, Paisley, United Kingdom).

Initially a total of 31 SNPs in the metabolic pathway of vitamin D were selected on basis of previously shown association with serum 25OHD concentration. All SNPs that associated (p<0.05) with serum 25OHD concentration in all mothers (mothers of type 1 diabetic and non-diabetic children as one group) in our previous study [40], were selected for further analyses (13 SNPs). Five SNPs in *NADSYN1/DHCR7* (rs4945008, rs12785878, rs7944926, rs3794060, rs12800438) and two SNPs in the *VDR* (rs731236 and rs154410) were in strong linkage disequilibrium and thus had similar associations with serum 25OHD concentration. Results of only one SNP of these SNP groups are presented resulting in the final number of 7 SNPs in the present study.

### Serum 25OHD concentrations

Serum 25OHD concentration measurement has been described in detail in our previously published study [17]. Briefly, serum 25OHD concentration was determined with an enzyme immunoassay method with IDS OCTEIA 25-Hydroxy Vitamin D kit, (Immunodiagnostic Systems Ltd., Boldon, UK). The intra- and inter-assay CVs were 3.57% and 3.68%, respectively. The analytical reliability of 25OHD assay was assured by participation in the vitamin D External Quality Assessment Scheme i.e. DEQAS (Charing Cross Hospital, London UK).

### Statistical methods

Statistical analyses were performed using Intercooled Stata10 for Windows (StataCorp. 2007. *Stata Statistical Software*: *Release 10*. College Station, TX: StataCorp LP) and SAS 9.3 (SAS Institute Inc., Cary, NC). Linear regression modelling was used to analyse the effect of presence of an allele in each SNP on the serum 25OHD concentration. Due to the known seasonal difference in serum 25OHD concentrations on Finland, all analyses were adjusted for the month of sample collection. In order to test the difference between mothers of type 1 diabetic and mothers of non-diabetic children, interaction term between the two mother types and allele dosage in each SNP was incorporated into the models. The interaction was significant in the SNPs rs12512631 and rs4516035 indicating a difference in the effect of allele dosage for mother type. Therefore in the second stage the effect of the presence of C allele in rs12512631 and T allele in rs4516035 on the serum 25OHD concentrations were studied separately in mothers of type 1 diabetic and non-diabetic children, and seasonally adjusted mean serum 25OHD concentrations were estimated.

## Results

Differences in the strength of the association between serum 25OHD concentration and two SNPs in the metabolic pathway of vitamin D were found. These SNPs associated stronger with serum 25OHD concentration during pregnancy in mothers of type 1 diabetic children than in mothers of non-diabetic children.

**Fig 1:**
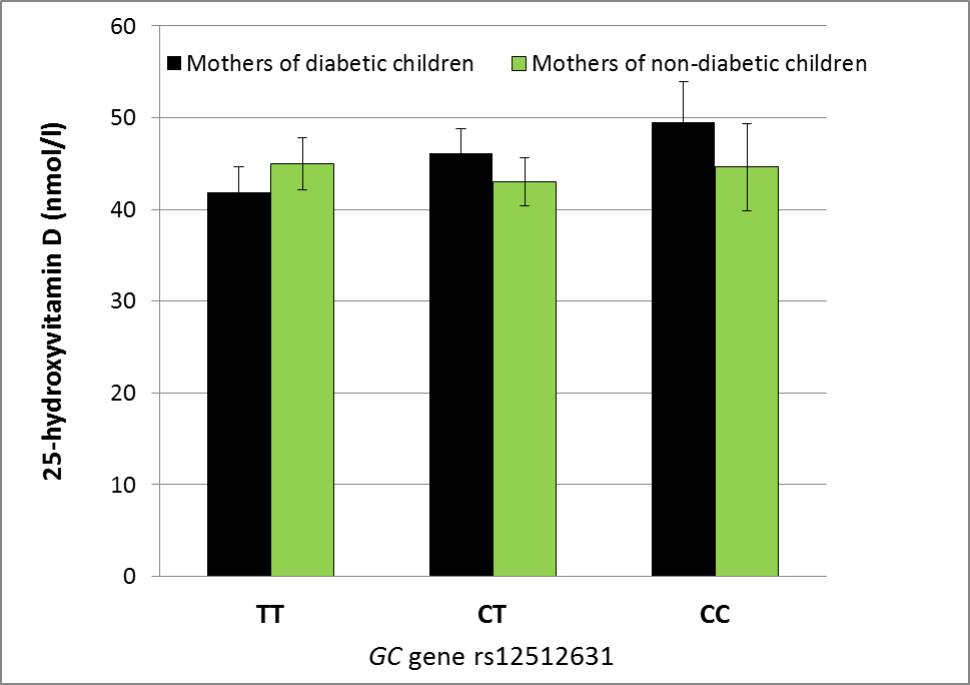
In mothers of type 1 diabetic children the presence of C allele in the genotype is associated with an average difference of 3.9 nmol/l in serum 25OHD concentration (*p* = 0.002) while in mothers of non-diabetic children such an association was not found (*p* = 0.64) (*p*_interaction_ = 0.02). All analyses were adjusted for the month of sample collection.

In mothers of type 1 diabetic children the presence of each C allele of rs12512631 located in the *GC* gene was associated with a rise of 3.9 nmol/l in serum 25OHD concentration (*p* = 0.002; i.e. CC genotype compared with CT +3.9 nmol/l and with TT +7.8 nmol/l). In mothers of non-diabetic children the mean serum 25OHD concentrations between the different genotypes of rs12512631 were similar (*p* = 0.64) (*p*_interaction_ = 0.02) (Table 1, Fig 1).

**Table 1:** Association of 25-hydroxyvitamin D concentration with SNPs in the metabolic pathway of vitamin D in all mothers and separately for mothers of type 1 diabetic and non-diabetic children, and according to the presence of the effect allele (EA). All analyses were adjusted for month of sample collection.

SNP	Gene	All mothers	Mothers of type 1 diabetic children	Mothers of non-diabetic children	Effect of presence of the effect allele			
		*p* (n)	*p* (n)	*p* (n)	EA*	*p*_*Cases*_	*p*_*Controls*_	*p*_interaction_
rs4945008	*NADSYN1/DHCR7*	0.03 (766)	0.20 (445)	0.05 (325)	G	0.09	0.03	0.89
rs4516035	*VDR* Prom	0.02 (764)	0.004 (445)	0.93 (321)	T	0.0002	0.83	**0.03**
rs1544410	*VDR* Bsm1	0.03 (747)	0.09 (427)	0.32 (326)	A	0.04	0.20	0.58
rs10783219	*VDR*	0.02 (751)	0.04 (437)	0.55 (317)	T	0.008	0.35	0.23
rs12512631	*GC*	0.03 (755)	0.02 (435)	0.67 (324)	C	0.002	0.64	**0.02**
rs4588	*GC*	0.05 (737)	0.05 (419)	0.65 (322)	C	0.02	0.50	0.26
rs17470271	*CYP27A1*	0.03 (750)	0.05 (428)	0.49 (325)	T	0.03	0.86	0.21

*The effect allele (EA) is the allele that increases the 25-hydroxyvitamin D concentration

**Fig 2:**
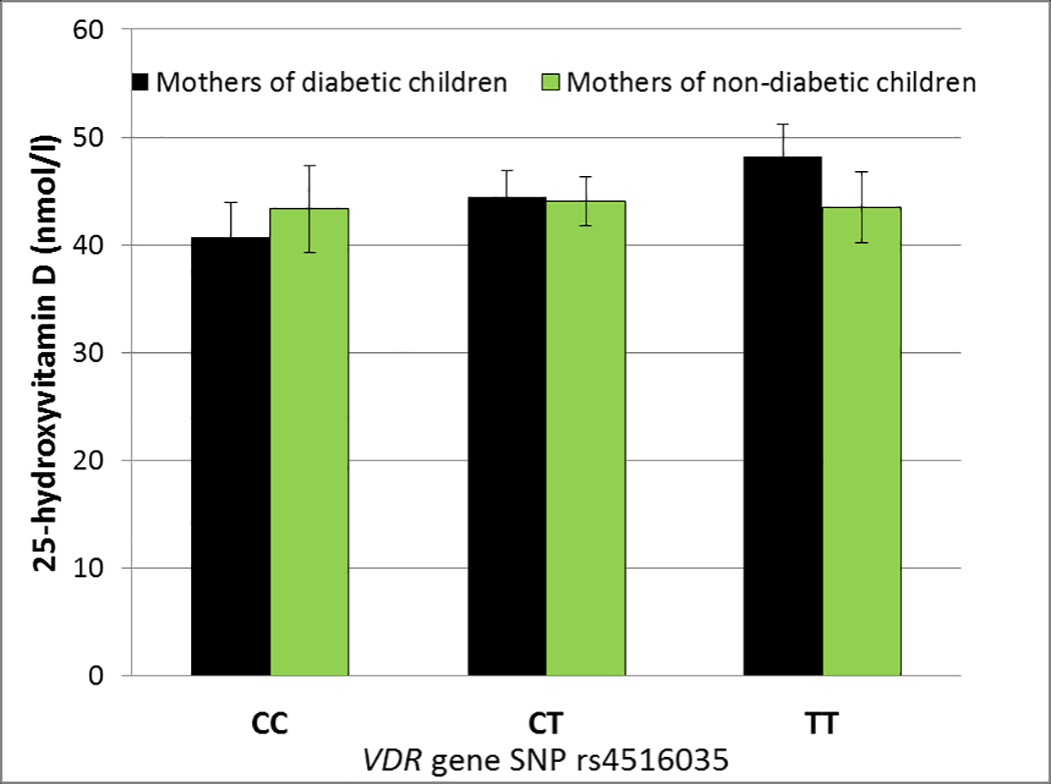
In mothers of type 1 diabetic children the presence of T allele is associated with an average difference of 4.1 nmol/l l in serum 25OHD concentration (*p* = 0.0002) while in mothers of non-diabetic children such an association was not found (*p* = 0.83) (*p*_inetraction_ = 0.03). All analyses were adjusted for the month of sample collection.

Similarly, in mothers of type 1 diabetic children the presence of each T allele in the genotype of rs4516035 located in the promoter region of the *VDR* gene was associated with a rise of 4.1 nmol/l in serum 25OHD concentration (*p* = 0.0002; i.e. TT genotype compared with CT +4.1 nmol/l and with CC +8.1 nmol/l), while in mothers of non-diabetic children the mean serum 25OHD concentrations between the different genotypes of rs4516035 were similar (*p* = 0.83) (*p*_interaction_ = 0.03) (Table 1, Fig 2).

Including mother´s age in the statistical model did not change the conclusion. Also, the associations between serum 25OHD concentration and SNPs were similar in strength between younger and older mothers.

## Discussion

The existing evidence of an association between vitamin D and type 1 diabetes is inconsistent. Identification of vitamin D metabolism or vitamin D genetics-related differences between patients and controls and between their mothers will help revealing the mechanism explaining the possible association. We found for the first time that during pregnancy the associations between serum 25OHD concentration and two polymorphisms in the *VDR* and *GC* genes are stronger in mothers of type 1 diabetic children than in mothers of non-diabetic children.

We had the possibility to select the study groups and use samples collected from the mothers during pregnancy, when already known which of the children later developed type 1 diabetes. We were therefore able to retrospectively investigate differences between mothers of type 1 diabetic and non-diabetic children or between pregnancies of the mothers.

Stronger associations during pregnancy of mothers of type 1 diabetic children between serum 25OHD concentration and SNPs were seen in two out of four gene loci that were studied. This decreases the possibility of false positive findings. Nevertheless, our results need to be confirmed. In our study vitamin D status was available only from the mothers. In the future it should be investigated whether the genetic control of serum 25OHD concentrations differs also between type 1 diabetic patients and controls.

No previous studies exist with a similar study setting. However, in a Finnish Population-based health survey (FIN-D2D 2007), it was recently found that age and possibly also gender modify the strength of associations between serum 25OHD concentration and SNPs in the metabolic pathway of vitamin D. One of the SNPs that showed difference in strength of an association with serum 25OHD concentration between younger and older adults in the FIN-D2D Health Survey 2007, was the same that showed difference in strength of an association with serum 25OHD concentration between mothers of type 1 diabetic children and mothers of non-diabetic children in the present study (rs12512631 in the *GC*).

In the search for genetic risk factors for type 1 diabetes, the main focus has been on the patient. The human leukocyte antigen (HLA) region accounts for most of the genetic risk and genome-wide association studies (GWAS) have identified over 50 additional genetic regions that also affect the risk for developing type 1 diabetes [41].

However, the *in utero* environment seems also to contribute in the risk for type 1 diabetes. High birthweight, high maternal age or infections during pregnancy have associated with an increased risk for type 1 diabetes in the child [6–10]. Maternal gluten-free diet has been shown to reduce diabetes incidence in the offspring of NOD mice [42]. In our previous study it was shown that mothers of type 1 diabetic and mothers of non-diabetic mothers have genetic differences in the VDR gene, irrespective of the corresponding genetic variants in the patients and control children [40]. Since the *VDR* is known to regulate the expression of at least 500 genes [39], some of these genes may influence the development of the immune system and therefore early programming of type 1 diabetes. Thus, the stronger association between SNPs and serum 25OHD concentration in mothers of type 1 diabetic patients that was seen in the present study may not associate directly with type 1 diabetes, but may be a marker of a difference in vitamin D metabolism and /or in the function of the *VDR*.

The fact that strength of genetic association of a certain polymorphism can vary considerably between subgroups stratified according to life-style factor or host characteristics has not yet been thoroughly studied. It has been shown though, that several polymorphisms associate stronger with fat distribution in women than in men [43]. Also, a certain polymorphism has been shown to associate with juvenile idiopathic arthritis only in women [44]. Several polymorphisms have shown stronger associations with body mass index in younger than older people [45]. Smoking can change the direction of a genetic association: A certain polymorphism has been found to associate with low body mass index in smokers, but with high body mass index in non-smokers [46]. These results suggest that part of the associations cannot be detected in GWAS [47] and therefore, the genetic differences between subpopulations may prove to be highly significant when searching for the genetic determinants of a disease or physiological trait.

We show for the first time that there are differences in the strength of genetic determinants of serum 25OHD concentration during pregnancy between the mothers of type 1 diabetic and non-diabetic children. Our results emphasize that the in utero environment including maternal vitamin D metabolism should be important lines of investigation when searching for factors that lead to early programming of type 1 diabetes.
